# Hints on the Treatment of Errors of Refraction

**Published:** 1907-07-06

**Authors:** 


### Ophthalmology.

### HINTS ON THE TREATMENT OF ERRORS OF REFRACTION.

### The Apparatus Required.

For testing visual acuity and for the estimation of errors of refraction a well-lighted room is required, which if possible should be twenty feet long. The modern system of visual notation supposes that the test is carried out with the patient six metres, or approximately twenty feet, from the types which he is required to read. Twenty-five feet is a more desirable length for the room, as it allows for a chair on which the patient may sit, and gives a space behind the chair for the oculist to move to and fro without crossing the line of vision.

Should the room be less than fifteen feet long, it may be arranged that the patient shall see types placed above his own head and viewed in a mirror on the opposite wall. This doubles the apparent distance of the types, and under these conditions a twenty foot basis may be obtained in a room with a diagonal of little over ten feet. The types seen in a mirror are, of course, reversed, and special cards with reversed types must be obtained for the purpose. The letters should properly be illuminated by artificial light, as a more constant standard is obtained, though a good top or north light falling directly upon the types is almost as satisfactory. The letters should be mounted on wood, or painted black upon white opaline glass, and should range in size from $\frac{6}{60}$ to $\frac{6}{5}$.

For retinoscopy and the examination of the fundus oculi a good Argand gas burner is the best light, but should this be not available an incandescent mantle in a metal chimney with a frosted-glass aperture, an incandescent electric globe, with a filament in the form of a gridiron instead of a loop, or a broad-flamed oil lamp are fairly efficient substitutes. Any light for ophthalmic purposes should be mounted upon an arm which has an elbow joint, and can be moved up and down.

### The Test Lenses.

The lenses required for testing purposes are the most expensive part of the outfit. It is necessary to have a complete double set of both convex and concave glasses from .25D to 20D, with double sets of cylinders from .25D to 6D. In addition, a set of prisms will be required, and also coloured glasses, diaphragms, and spectacle frames with two grooves, for spherical and cylindrical glasses at the same time. Contracted or portable sets of lenses, with simplified spectacle frames, are useless, as so much time is lost in making up the proper combinations of lenses, and the vision is never so accurately estimated through three or four glasses as through two only. The lenses can be obtained either mounted or unmounted; the former costing about £13, the latter about £10.

For retinoscopy alone a simple plane mirror is advised, though the ordinary concave mirror of the ophthalmoscope may be used for the purpose. The advantages of a plane mirror are that it is more accurate in its estimation, that its accuracy does not vary with the distance of the operator from the patient, that it is larger and so gives more light, and that it can also have a larger central aperture, which should not be less than $\frac{1}{6}$ in. in diameter. An ophthalmoscope also will be required for further investigations into ocular defects, but as this instrument forms part of every doctor's outfit no further reference need be made to it.

### Routine.

It is advisable to do a retinoscopy upon every patient who may wish to be tested for glasses. All children should be treated before retinoscopy by the instillation of atropine drops (four grains of the sulphate to 1 oz. of water) into both eyes three times a day for a week. Children are notoriously tolerant of atropine, and it is only in very occasional cases that dryness of the throat and thirst, and more rarely still that sleeplessness and nocturnal delirium occur.

All young adults should also be treated with atropine or homatropine. As their time is more valuable and the effects of atropine take some week or ten days to wear off, it is usually better to use homatropine or a combination of homatropine and cocaine. The following formula makes the best solution:—

| | | |
|---|---|---|
| Homatropine hydrobromate | ... ... | gr. iv. |
| Cocaine hydrochloride | ... ... ... | gr. viij. |
| Distilled water to | ... ... ... | 1 oz. |

As homatropine is a very expensive drug, it is advisable not to order it for patients unless absolutely necessary, for 1 oz. of such a solution as this costs anything from 6s. to 12s. It is the most convenient for the practitioner to keep in stock, as its action is very complete and rapid, but as the solutions do not keep very well, those who are likely to need homatropine and cocaine but seldom are advised to get lamellæ or ophthalmic tablets.

If homatropine and cocaine are used the drops should be put into the patient's eyes at least twice, at an interval of ten minutes. At the end of another twenty or thirty minutes the pupils will be thoroughly dilated, and the accommodation, though not paralysed entirely, will be much lessened in power.

Patients over forty should in ordinary cases be tested for glasses without dilatation of pupil. It is usually required to test their vision for near as well as distant vision, and the use of mydriatics will prevent this being done.

In difficult cases of astigmatism, however, or failure of vision from defects of the media or the retina, dilatation of the pupil is required. This can be done quite safely if due precautions are taken to avoid cases of increased tension; homatropine and cocaine is the most useful combination for the purpose.

